# Application of Exogenous Iron Alters the Microbial Community Structure and Reduces the Accumulation of Cadmium and Arsenic in Rice (*Oryza sativa* L.)

**DOI:** 10.3390/nano12081311

**Published:** 2022-04-11

**Authors:** Tingting Li, Jiayuan Li, Xin Zhan, Xueli Wang, Bing He, Feishu Cao, Changjun Liao, Yuefeng Yu, Zengyu Zhang, Junhui Zhang, Bei Li, Jiancheng Chen, Hong Li, Zhiqiang Zhu, Yanyan Wei, Junming Hu

**Affiliations:** 1 State Key Laboratory for Conservation and Utilization of Subtropical Agro-Bioresources, Cultivation Base of Guangxi Key Laboratory for Agro-Environment and Agro-Products Safety, College of Agriculture, Guangxi University, Nanning 530004, China; ltt1210@126.com (T.L.); 1917303005@st.gxu.edu.cn (J.L.); njueczx@163.com (X.Z.); wxl0524@126.com (X.W.); bingh2000@126.com (B.H.); 1817303017@st.gxu.edu.cn (Z.Z.); le_bei@st.gxu.edu.cn (B.L.); 2017392001@st.gxu.edu.cn (J.C.); 2 Agricultural Resources and Environment Research Insititute, Guangxi Academy of Agricultural Sciences, Nanning 530007, China; yuyue202204@163.com (Y.Y.); zjh914zjh914@163.com (J.Z.); 3 Guangxi Bocco Enviromental Protection Technology Co., Ltd., Nanning 530007, China; feishu.cao@hotmail.com (F.C.); lcj19176042408@163.com (C.L.); 4 Key Laboratory of Eco-Environment of Three Gorges Region, Ministry of Education, Chongqing University, Chongqing 400044, China; hongli@cqu.edu.cn; 5 College of Tropical Crops, Hainan University, Haikou 570228, China; zqzhu@hainanu.edu.cn

**Keywords:** cadmium, arsenic, rice, microorganism, ferrous sulfate, ferric oxide nanoparticle

## Abstract

Cadmium (Cd) and arsenic (As) contamination of soil has been a public concern due to their potential accumulation risk through the food chain. This study was conducted to investigate the performance of ferrous sulfate (FeSO_4_) and ferric oxide (Fe_2_O_3_) nanoparticle (Nano-Fe) to stabilize the concentrations of Cd and As in paddy soil. Both Fe treatments led to low extractable Cd and the contents of specifically sorbed As contents, increased (*p* < 0.05) the Shannon index and decreased (*p* < 0.05) the Simpson diversity indices compared with the control. Nano-Fe increased the relative abundances of Firmicutes and Proteobacteria and decreased the abundances of Acidobacteria and Chloroflexi. Moreover, the addition of both forms of Fe promoted the formation of Fe plaque and decreased the translocation factor index (TFs) _root/soil_, TFs _shoot/root_, and TFs _grain/shoot_ of Cd and As. These results suggest that exogenous Fe may modify the microbial community and decrease the soil available Cd and As contents, inhibit the absorption of Cd and As by the roots and decrease the transport of Cd and As in rice grains and the risk intake in humans. These findings demonstrate that soil amendment with exogenous Fe, particularly Nano-Fe, is a potential approach to simultaneously remediate the accumulation of Cd and As from the soil to rice grain systems.

## 1. Introduction

Cadmium (Cd) and arsenic (As) are common toxic elements in soil and are classified as the number one carcinogens by the International Agency for Research on Cancer [1,2]. Recent nationwide surveys show that the rates of Cd and As in China are 7.0% and 2.7%, respectively, which are higher than China’s soil environmental quality limits [3]. Co-pollution with Cd and As in the paddy soil of South China is particularly serious owing to the rapid development of urbanization and mineral production and processing [4]. The excessive intake of Cd can lead to Cd poisoning, which can cause kidney poisoning and chondroplasty [5]. As has a clear causal relationship with the occurrence of skin and lung cancer and is closely related to the occurrence of visceral cancer, such as liver and bladder cancers. Therefore, reducing the amount of Cd and As is of the utmost importance.

Rice is a major grain, which is the main food source of more than half of the global population [6], and is also the main food source of Cd and As in many Asian countries, particularly in China [6]. Cd and As are difficult to control simultaneously because their geochemical behavior and bioavailability differ in the paddy field environment owing to the alternation of soil that is dry and wet [7,8]. The soil redox potential has a substantial influence on the bioavailability of Cd and As. Drainage and flooding cycles in paddy fields can change the soil redox potential, thus, affecting the fate of Cd and As [9]. Owing to the need for water-logging treatment in the process of planting rice under reducing conditions, Cd can form insoluble precipitates with sulfur, which reduces their availability. However, As in the soil transforms into a soluble state, and its bioavailability is enhanced. It is absorbed by the rice plants through their roots. Under oxidation conditions, the solubility of Cd increases owing to the inhibition of sulfur reduction, while the availability of As decreases as the content of Fe(Ⅲ) oxide increases [6]. Thus, the simultaneous reduction of Cd and As uptake in paddy soil is more challenging than the control of a single pollutant.

To diminish the impact of Cd and As pollution on ecosystems and agriculture, researchers have proposed various biological, physical, and chemical procedures [7], such as soil replacement, leaching, and phytoremediation [10,11,12]. However, for agricultural land with a large area and a low amount of pollution, these technologies take a long time to repair and improve soil quality, are expensive, easily cause secondary pollution and often destroy the structure of soil microbial community [13,14]. In situ immobilization with a passivator is a widely studied technique because of its high efficiency, low cost, and easy operation [8]. Passivation materials include clay minerals, Fe compounds, and biochar [10], among those that remediate Fe are particularly attractive. The carbon-containing complex with Fe compounds reduced the adsorption of Cd and As in aqueous solutions [15,16]. Some studies used materials modified with Fe in the soil. Namely, a solution of 3.0% (mass ratio) ferrous sulfate-modified nano-silica designated RNS-SFe immobilizes the bioavailable Cd and As in the soil by 85.0% and 80.1%, respectively, by transforming the bioavailable Cd into insoluble mercapto metal compounds (eS-Cd-S-) and bioavailable As into less soluble iron arsenate (Fe_3_[AsO_4_])_2_ and FeAsO_4_), which precipitated on the surface of nano-silica particles [17]. Fe-enriched corncob-eggshell biochar significantly reduced the amounts of Cd and As in brown rice by affecting the rhizosphere soil pH and redox potential (Eh) and causing the formation of Fe-plaque [18]. Some studies also found that biochar modified with Fe could not simultaneously reduce Cd and As. Biochar could reduce the concentrations of Cd in the roots (49–68%) and grains (26–49%), but this results in a simultaneous increase of As concentrations in roots [19]. In contrast to treatment with biochar, 0.5% Fe-biochar decreased the As concentrations in the roots, and also increased the concentrations of Cd. The mobilization of induction of soil Cd by Fe-biochar was probably owing to the acidification of the soil [19]. In addition, as a new environmental-friendly material for heavy metal remediation, iron nanoparticles has attracted great attention [20]. According to the analysis above, the most closely related system described in the literature was the soil-rice system. They mainly used iron solution modified materials to study the morphological transformation and plant cumulative absorption of Cd and As in the soil-rice system. Nevertheless, little information is available on the effect of exogenous Fe materials, particularly Fe oxide nanoparticles (Nano-Fe), on the bioavailability and transportation of Cd and As in the soil-rice system and possible microbial responses.

In this study, a pot experiment was conducted to explore the effects of ferrous sulfate and Nano-Fe applications on (i) rice growth; (ii) Cd and As speciation and the microbial community in soil; (iii) sequestration of Cd and As by root Fe-plaque; and (iv) the uptake and intake risk assessment of Cd and As in paddy rice. The purpose of this experiment was to improve the theoretical basis for the remediation of heavy metal(loid)s and to provide a new way for sustainable development management and food security of rice fields polluted with Cd and As.

## 2. Materials and Methods

### 2.1. Site Description

The rice pot experiment was conducted in the greenhouse of the Institute of Agricultural Resources and Environment, Academy of Agricultural Sciences, Nanning City, Guangxi, China (22°51′ N, 108°15′ E). The soil used in the rice pots was collected from the surface soil (0–20 cm) polluted by Cd and As in Jinchengjiang, Hechi, Guangxi, China. Soil properties include a soil pH of 6.80, organic matter content of 30.40 g·kg^−1^, total N content of 1.90 g·kg^−1^, available K content of 327.00 mg·kg^−1^, total Cd content of 1.73 mg·kg^−1^, and total As content of 106.93 mg·kg^−1^. The rice variety was indica type three-line late maturing hybrid rice Yixiang 99E-4.

### 2.2. Experimental Design and Sampling

The experiment was conducted using a completely randomized design. Three treatments were established in this experiment: (1) control; (2) 0.50 g·kg^−1^ of ferrous sulfate added (FeSO_4_); and (3) 0.50 g·kg^−1^ of nano iron oxide added (Fe_2_O_3_) (Nano-Fe). Nano-Fe was shown to possess a negatively charged surface with a zeta potential value of 13.40 ± 0.26 mV. The size and morphology of nano-Fe were studied by scanning electron microscopy (SEM) (TESCAN Inc., Brno, Czech Republic) (Figure S1). X-ray energy spectrum analysis (EDS) (TESCAN Inc., Brno, Czech Republic) and Fourier transform infrared spectroscopy (FTIR) (Thermo Inc., Waltham, MA, USA) of Nano-Fe are shown in Figures S2 and S3, respectively. There were three repetitions for each type of treatment. After natural air drying, the soil was screened for 2 mm to remove large stones and plant stalks. Iron material and base fertilizer were mixed with 8.0 kg soil in a basin (30 cm × 33 cm, d × h). The soil was soaked in tap water for 8 d to ensure that the ions were fully dissolved and mixed. The rice seeds were sterilized with 5.0% sodium hypochlorite for 5 min, soaked in tap water for 8 h, and placed in a dark and humid environment for 48 h. Rice with approximately 1.50 cm high was transferred to the greenhouse of Guangxi University and cultured in the substrate. When the rice had three leaves of the same height, they were transplanted into the basin under ambient air and sunlight conditions from early April to mid-July, within 101 d. Three rice plants were evenly distributed in each basin, and two plants were inserted into each point. The position of the pots was changed every 5 d. The water level in each basin was controlled at approximately 2.0 cm from the soil level. The dosages of N (urea), P (KH_2_PO_4_), and K (KCl) were 0.1304 g·kg^−1^ (base fertilizer: 0.0391 g·kg^−1^, tiller fertilizer: 0.0391g·kg^−1^, ear fertilizer: 0.0522 g·kg^−1^), 0.4394 g·kg^−1^, and 0.2325 g·kg^−1^, respectively.

Soil and plant samples were collected when the rice had matured on July 15. On the day of sampling, fresh rice rhizosphere soil samples were removed with sterilized glass rods, placed in 5 mL centrifuge tubes, sealed, and placed in a tank of liquid nitrogen. Soil samples were sent to Shanghai Meiji Biomedical Technology Co., Ltd. (Shanghai, China) for high-throughput sequencing to explore the changes in composition of total bacteria (16S rRNA) in different soil treatments. In addition, the soil samples after air drying and screening were analyzed for soil properties, including the contents of different forms of Cd and As. Samples of Fe plaque on the surfaces of rice roots were extracted to determine the concentrations of Fe, Cd, and As on the root surfaces. The plant samples were washed twice with tap water, treated with ultrapure water, dried at 105 °C for 30 min, and then dried to a constant weight at 65 °C. The roots, shoots, and grains yield were measured by weighing after drying, and they were cut and ground. They were screened through a 0.15-mm sieve and bagged to determine the concentrations of Cd and As.

### 2.3. Soil Chemical Analysis

The soil pH was measured by a pH meter (Leici Inc., Shanghai, China) with a glass electrode using a ratio of 1:2.5 (*w*/*v*). The soil organic matter was determined by a potassium dichromate sulfuric acid external heating method. The Kjeldahl method was used to determine the soil total N. A volume of 1 mol·L^−1^ N_4_OAc was used as the extractant. The soil was shaken for 0.5 h and filtered, and the contents of soil available K were determined directly by flame photometry (Xinyi Inc., Shanghai, China). The contents of total soil Cd were determined after soil digestion by HF-HClO_4_-HCl using an atomic absorption spectrometer (Hitachi Inc., Tokyo, Japan) [21]. The total soil As content was determined by acid digestion (HNO_3_, HCl and HF) using an atomic fluorescence spectrometer (Titan Inc., Beijing, China) [22].

A four-part continuous extraction method [23] was used to separate the Cd from the samples into four forms, e.g., exchangeable Cd, organically bound Cd, inorganically bound Cd, and residual Cd. The extraction solutions were determined by atomic absorption spectrometry (Hitachi Inc., Tokyo, Japan).

A five-part continuous extraction method [24] was used to separate the As from samples into five forms, e.g., non-specifically sorbed As, specifically sorbed As, amorphous iron oxide As, Fe/Al hydrated oxide As, and residual As. The extraction solutions were determined by atomic fluorescence spectrometry (Titan Inc., Beijing, China).

### 2.4. Analysis of Fe, Cd, and As on the Roots Surfaces

Fresh roots (10.0 g) were cleaned with deionized water and weighed into plastic bottles to determine the concentrations of Fe, Cd, and As on the roots surfaces. DCB extractant (90 mL of 0.30 mol·L^−1^ sodium citrate and 10 mL of 1.0 mol·L^−1^ sodium bicarbonate) and approximately 3.0 g of sodium bicarbonate were added in turn, and shaken at 180 rpm for 2 h in a full temperature shaking incubator. After that, the concentrations of Fe, Cd, and As in the extractant were determined by atomic absorption spectrometry (Hitachi Inc., Tokyo, Japan). The extracted rice roots were dried in an oven, and were weighed to determine their dry weight. The concentrations of Fe, Cd, and As on the roots surfaces were calculated (mg·kg^−1^ of dry root) [25].

### 2.5. Analysis of Cd and As in the Roots, Shoots, and Grains

A microwave digestion method was used to measure the concentrations of Cd and As in the roots, shoots, and grains using 0.20 g of plant samples. Each tissue sample was weighed in a digestion tube, and 5.0 mL of concentrated nitric acid was added. A parallel standard sample and a blank were also created, and then microwave digestion (500 power, 150 °C, and 8 min) was conducted. The digested samples were removed and placed on an acid driving rack to drive the acid (120 °C) to the size of a soybean granule. The samples were cooled and transferred to a 25 mL volumetric flask. Finally, the samples were filtered into a plastic bottle. The content of Cd was measured by atomic absorption spectrometry (Hitachi Inc., Tokyo, Japan), and the content of As was measured with an atomic fluorescence spectrometer (Titan Inc., Beijing, China).

### 2.6. qPCR Amplification and High-Throughput Sequencing of the Soil Bacteria (16S rRNA)

The microbial community genomic DNA in soil was extracted using an E.Z.N.A.^®^ soil DNA Kit (MP Bio-tek, Norcross, GA, USA). The primers 338F (5’-ACTCCTACGGGAGGCAGCAG-3’) were used to amplify the V3-V4 hypervariable region of the 16S rRNA gene. The PCR amplification was performed as follows: initial denaturation at 95 °C for 3 min, 27 denaturing cycles at 95 °C for 30 s, annealing at 55 °C for 30 s, extension at 72 °C for 45 s, single extension at 72 °C for 10 min, and end at 4 °C. As described in the standard protocols of Majorbio Bio-Pharm Technology Co. Ltd. (Shanghai, China), purified amplicons were pooled in equimolar amounts of paired-end sequenced (2 × 300) on an Illumina MiSeq platform (Illumina, San Diego, CA, USA). Operational taxonomic units (OTUs) with a 97% similarity cutoff were clustered by using UPARSE (version 7.1, http://drive5.com/uparse/, accessed on 19 June 2021), and chimeric sequences were identified and removed by using UCHIME. The taxonomy of each OTU representative sequence was analyzed by RDP Classifier (http://rdp.cme.msu.edu/, accessed on 19 June 2021) against the 16S rRNA database (e.g., Silva SSU128) with a confidence threshold of 0.7.

### 2.7. Data Processing and Analysis

The health risk index (*HRI*) of Cd and As was calculated by estimating the daily intake of Cd (*DIM*) and As (*DIM*) and with an oral reference dose of *RFD* of Cd and As. The *RFD* values of Cd and As were considered to be 0.001 mg·kg^−1^ and 0.0003 mg·kg^−1^, respectively. Bodyweight day^−1^ was determined as previously described [26].

(1) HRI=DIM/RFD

(2) $$DIM=Cmetal*F*\left(D\;food\;intake\right)/\left(Baverage\;weight\right)$$

where *C_metal_* indicates the accumulation of metal in the grain (mg·kg^−1^); F indicates the conversion factor (0.085); *D_food intake_* indicates the daily use of grains considered to be 0.40 kg per person, and *B_average weight_* indicates the body average weight, which was considered to be 70.00 kg per person.

The translocation factor index (TF) was determined as described by Singh et al. [27]. The experimental data were analyzed by SPSS 21.0 (IBM Inc., Armonk, NY, USA) and compared by Duncan’s method. GraphPad Prism 7.0 (GraphPad Inc., San Diego, CA, USA) was used for drawing.

## 3. Results

### 3.1. Yield of Various Parts of Rice

On the whole, compared with the control, the treatments with exogenous Fe (FeSO_4_ and Nano-Fe) increased (*p* < 0.05) the yield of shoots and grains, while only Nano-Fe treatment increased (*p* < 0.05) the root yields (Table 1).

### 3.2. Contents of Various Forms of Cd and As in the Soil

Exogenous Fe, in the form of FeSO_4_ and Nano-Fe, significantly decreased (*p* < 0.05) the contents of exchangeable, organically bound and inorganically bound Cd, which was more pronounced for Nano-Fe in comparison with FeSO_4_ (Table 2). Moreover, treatments of exogenous Fe increased (*p* < 0.05) the contents of Cd in the residual state (Table 2), decreased (*p* < 0.05) the specifically sorbed As, and increased (*p* < 0.05) the contents of Fe/Al hydrated oxide As (Table 3). However, only Nano-Fe decreased (*p* < 0.05) the amount of amorphous iron oxide As and increased (*p* < 0.05) the amount of residual As.

### 3.3. Concentrations of Fe, Cd, and As on the Roots Surfaces

**Figure 1:**
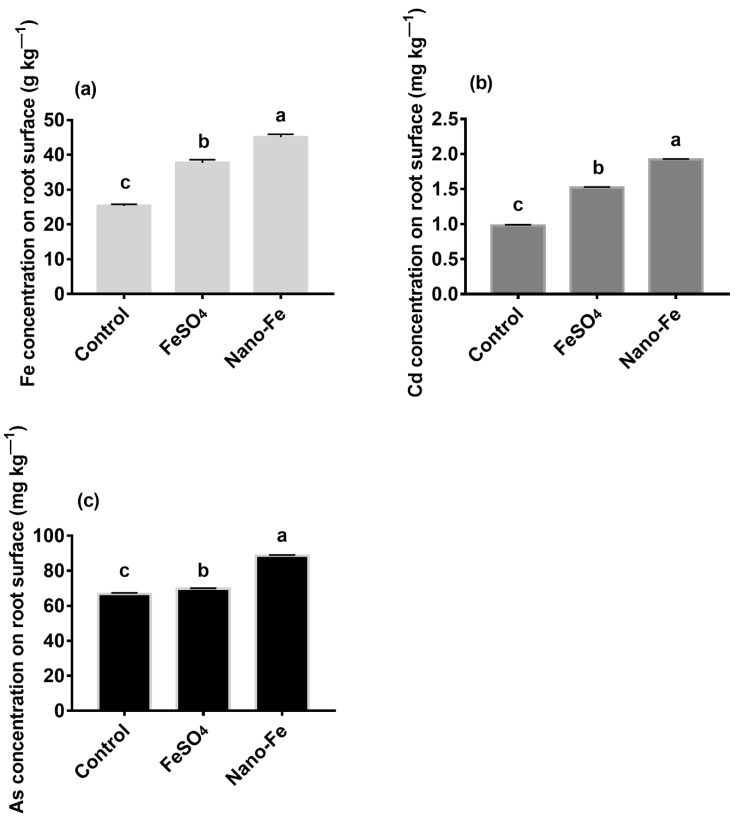
Concentrations of Fe (**a**), Cd (**b**), and As (**c**) on the surfaces of rice roots derived from treatments with exogenous Fe applied as FeSO_4_ or Nano-Fe. Different lowercase letters in the columns indicate a significant difference among the different treatments at *p* < 0.05.

With the addition of exogenous Fe, the concentration of Fe on the roots surfaces was higher (*p* < 0.05) than that in the control (Figure 1a). Moreover, FeSO_4_ and Nano-Fe also increased (*p* < 0.05) the concentrations of Cd and As on the roots surfaces, compared with the control (Figure 1b,c).

### 3.4. Concentrations, Uptake, and TFs of Cd and As in Rice

**Figure 2:**
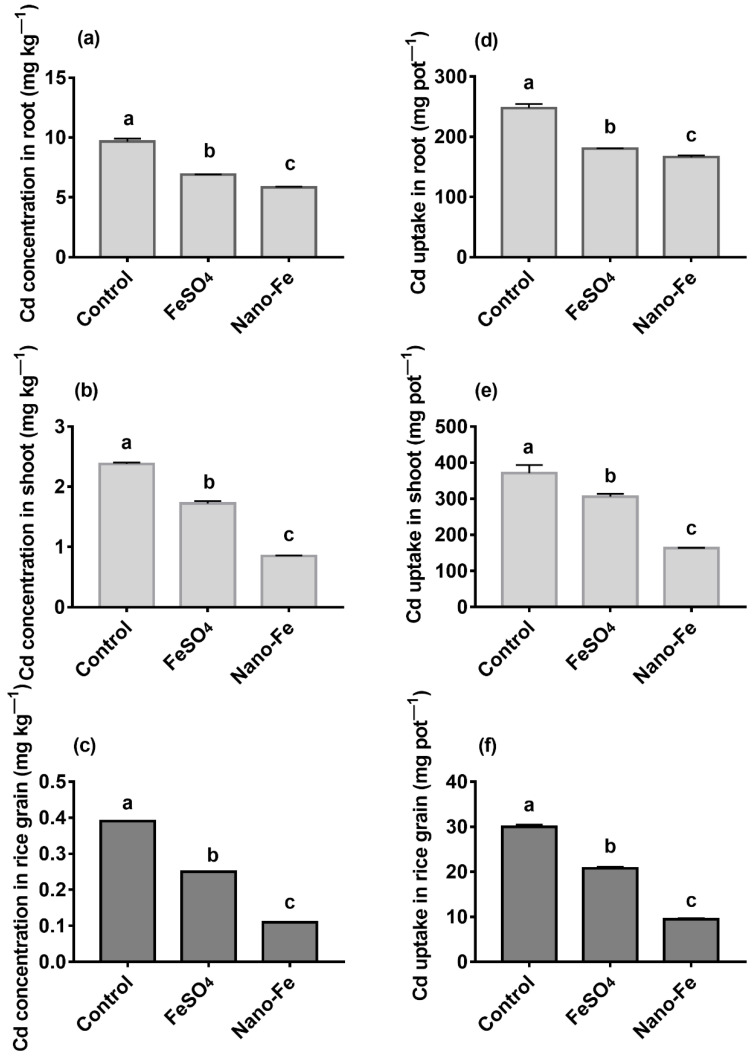
Concentrations of Cd in the roots (**a**), shoots (**b**), grains (**c**) of rice and uptake of Cd in the roots (**d**), shoots (**e**), grains (**f**) of rice derived from treatments with exogenous Fe applied as FeSO_4_ or Nano-Fe. Different lowercase letters in the columns indicate a significant difference among the different treatments at *p* < 0.05.

**Figure 3:**
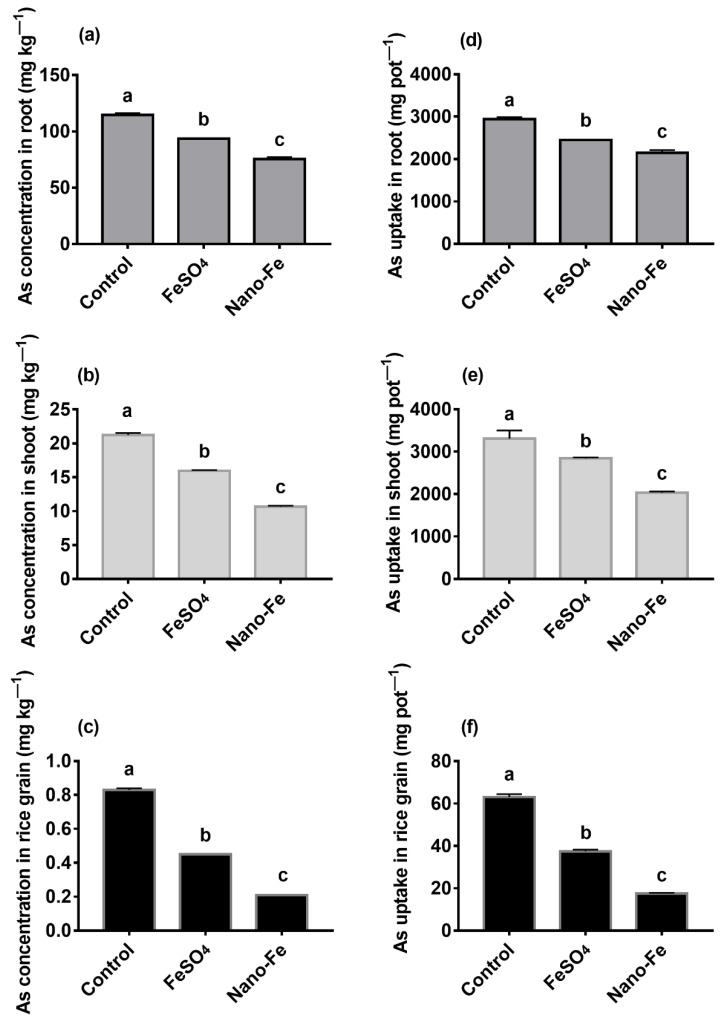
Concentrations of As in the roots (**a**), shoots (**b**), grains (**c**), and uptake of As in the roots (**d**), shoots (**e**), grains (**f**) of rice derived from treatments with exogenous Fe applied as FeSO_4_ or Nano-Fe. Different lowercase letters in the columns indicate a significant difference among the different treatments at *p* < 0.05.

Exogenous Fe reduced (*p* < 0.05) the Cd concentrations (Figure 2a–c) and its uptake (Figure 2d–f) in the roots, shoots, and grains, reduced (*p* < 0.05) the As concentrations (Figure 3a–c) and its uptake (Figure 3d–f) in the roots, shoots, and grains of rice, compared with the control.

**Figure 4:**
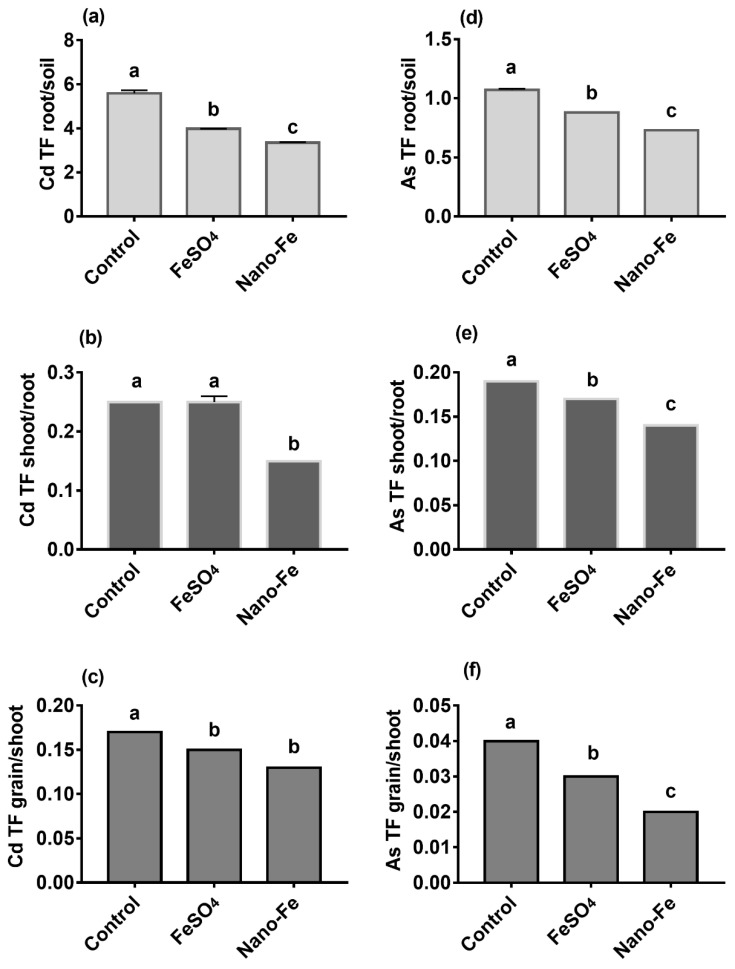
The Cd TF root/soil (**a**), shoot/root (**b**), grain/shoot (**c**), and As TF root/soil (**d**), shoot/root (**e**), grain/shoot (**f**) of rice derived from treatments with exogenous Fe applied as FeSO_4_ or Nano-Fe. Different lowercase letters in the columns indicate a significant difference among the different treatments at *p* < 0.05.

In terms of Cd, the soil to roots TFs were lower (*p* < 0.05) upon treatments with exogenous Fe, applied in both forms, compared with the control (Figure 4a), although this was more pronounced upon treatment with Nano-Fe. Compared with the control, the lower TFs from roots to shoots were recorded only upon exposure to Nano-Fe (Figure 4b). While both treatments with exogenous Fe equally lowered (*p* < 0.05) TFs values from shoots to grains (Figure 4b,c). In terms of As, the TFs from soil to roots, from roots to shoots and from shoots to grains were lower (*p* < 0.05) upon both treatments with exogenous Fe than those of the control (Figure 4d–f), although this effect was stronger in the Nano-Fe treatment.

### 3.5. Soil Microbial Community

**Figure 5:**
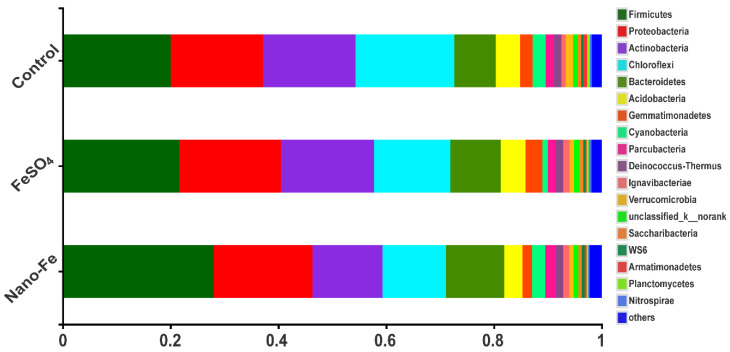
The relative levels of abundance of phyla in treatments with exogenous Fe applied as FeSO_4_ or Nano-Fe.

Treatments with exogenous Fe resulted in an increase (*p* < 0.05) in the Shannon diversity indices and a decrease (*p* < 0.05) in the Simpson indices compared with the control (Table 4). Firmicutes, Proteobacteria, Actinobacteria, and Chloroflexi were the dominant phyla and comprised 71.10–72.66% of the bacterial 16S rRNA gene sequences in all the treatments (Figure 5). The relative abundances of Firmicutes, Proteobacteria, Bacteroidetes, Gemmatimonadetes, Ignavibacteriae, unclassified_k__norank, and WS6 in the soil treated with FeSO_4_ were 12.17%, 7.99%, 10.51%, 21.45%, 33.42%, 46.69%, and 17.27% higher than those of the control, respectively. The relative abundances of Firmicutes, Proteobacteria, Bacteroidetes, Parcubacteria, and Planctomycetes in the soil treated with Nano-Fe were 39.88%, 7.37%, 40.82%, 30.26%, and 36.15% higher than those of the control, respectively. The treatments with exogenous Fe resulted in higher relative abundances of Firmicutes, Proteobacteria, and Bacteroidetes than those of the control (Figure 5).

### 3.6. Health Risk Assessment

Based on the *DIM* and *HRQ* formulas of the daily intake of Cd and As, the risk of dietary intake of Cd and As in rice with exogenous Fe was evaluated (Table 5). The results showed that the *DIM* values of Cd and As in rice polluted by Cd and As were 0.00019 and 0.00040 mg·(kg·D)^−1^, respectively. With the application of exogenous Fe, the *DIM* decreased, and the Nano-Fe was the lowest. The data showed that with the exogenous Fe, the *HRI* values of Cd and As were lower than those of the control, which ranged from 0.06 to 0.12 for Cd and from 0.34 to 0.73 for As. The *HRI* of Nano-Fe was the lowest among them. The *HRI* for Cd-As ranged from 0.40 to 0.85. Compared with the control, the *HRI* for Cd-As in rice decreased by 44–74% when exogenous Fe was applied. All of the *HRI* for Cd-As under the exogenous Fe treatments were <1 and met the safety limits.

## 4. Discussion

### 4.1. Speciation of Cd and As in Soil

Heavy metals in the soil can have different toxicities and environmental behavior owing to one or several aspects of the different fractions. The exchangeable component was the most mobile component in soil, which represents the bioavailability of organisms, while the residual phase was the lowest [28]. Based on the results of a continuous extraction experiment, the contents of residual Cd in the soil increased, and the contents of exchangeable Cd decreased after FeSO_4_ and Nano-Fe were applied to the soil (Table 2), which indicated that they could effectively reduce the migration rate and bioavailability of Cd in the soil. The reason could be that the addition of exogenous Fe to the soil could change the biogeochemical cycle of Fe. The application of FeSO_4_ can effectively stabilize Cd in the soil by surface precipitation and ion diffusion [29]. The application of Nano-Fe increased the contents of iron oxide/hydroxide, which was the most important factor that affected the mobility of Cd in the range of pH 5.0 to 7.5 [30].

Non-specific sorbtion is usually defined as the outer sphere, which represents the adsorption on the surface of soil minerals owing to the electrostatic attraction [31]. Specifically sorbed As forms a complex with coordinate covalent bonds [11]. Soil available As primarily originates from non-specific and specific sorption [31,32]. In this study, the addition of exogenous Fe did not affect the non-specifically sorbed As, and the contents of specifically sorbed As were significantly lower than that of the control, which was consistent with the results of previous research [32]. The addition of exogenous Fe promoted the increase in amount of Fe/Al hydrated oxide As and the residual As (Table 3). The reason could be that exogenous Fe can promote the diffusion of As from the outer surface of soil minerals to the inner surface and mineral lattice, resulting in the transformation of specifically sorbed As to other components, such as Fe/Al hydrated oxide and the residual fractions [33]. This indicated that exogenous Fe promoted the transformation of As in paddy soil from more mobile fractions to fewer mobile fractions, thus, reducing the uptake of As in the soil by rice.

Exogenous Fe has positive effects on soil microorganisms [34,35], which is the main driving force of the biogeochemical Fe cycle. Heavy metal pollution in soil usually leads to changes in the local microbial community [36]. The addition of exogenous Fe interferes with the effect of microbial community on pollution with Cd and As in the soil. Exogenous Fe altered the bacterial α-diversity and community structure. Nano-Fe resulted in the largest decrease in the contents of soil available Cd and As (Table 3 and Table 4). The relative abundances of phyla that help to reduce the phytotoxicity of Cd primarily included Firmicutes, which was consistent with the promotion of Firmicutes with the immobilization of Cd in soil [37]. Acidobacteria can reduce Fe (III) and Fe (II) concentrations [35]. The abundance of Acidobacteria decreased in Nano-Fe, which indicated that the reduction and oxidation of Fe were significantly weakened, which could be the reason why more available Cd was converted into residual Cd. Chloroflexi provides evidence of soil remediation [35]. The relative abundance of Chloroflexi in the Nano-Fe treatment was the lowest among all the treatments. At the phylum level, most of the Fe-oxidizing bacteria are members of Proteobacteria [38], and the increase of these bacteria in the Nano-Fe treatment could indicate that the oxidation of Fe (II) in the soil was enhanced. The change of As fractions in the soil may be due to treatment with Nano-Fe increased the relative abundance of Firmicutes and Proteobacteria in soil. Functional microorganisms can reduce As, dissimilatory As, As oxide, and methylated As, and also participate in nitrate, sulfate, and Fe reduction, which are considered to be resistant to As, as previously described [39,40]. Thus, exogenous FeSO_4_ and Nano-Fe applications in paddy soil could alter bacterial diversity and community structure, and increase heavy metals resistant bacteria.

In the soil-rice system, iron nanoparticles were applied to the soil, which increased the rice biomass (Table 1) and improved the soil microbial diversity (Table 4), so the iron nanoparticles were not toxic in our study. It is inconsistent with the results of other studies that iron nanoparticles are toxic [41]. The reason may be that the response characteristics of soil with different backgrounds and rice with different varieties to iron nanoparticles are different. In addition, an appropriate amount of iron nanoparticles is the key to affect the accumulation of heavy metals in rice [42,43].

### 4.2. Absorption of Cd and As in Rice

Rice roots are usually covered with Fe (III) oxide deposits, known as Fe plaque [44]. The structure of the Fe plaque is characterized by a mixture of crystalline and amorphous Fe (III) (oxy hydroxy) oxides, primarily in the form of Fe hydride and goethite [45]. The accumulation of Fe predicted Fe plaque formation. In this study, FeSO_4_ promoted the formation of Fe plaque (Figure 1a), which was consistent with the findings of Sebastian and Prasad [46]. The reason could be that anions such as SO_4_^2-^ attract H^+^ ions from the soil solids to soil solutions and form sulfuric acid, which affects the soil pH and forms Fe plaque [47]. The results show that the Fe concentrations in Fe plaque formed by Nano-Fe were higher than those of FeSO_4_ (Figure 1a). The formation of Fe plaque in treatments with FeSO_4_ primarily originated from Fe^2+^, while with Nano-Fe, the formation of Fe plaque primarily originated from the accumulation of nanometer Fe particles [44]. The Nano-Fe treatment, owing to the high level of adhesion and reactivity, resulted in nanoparticles that had a high specific surface area and adhesion to the epithelial root cell wall. Thus, they were more likely to form Fe plaque.

Fe plaque has been confirmed to limit the absorption of Cd and As [48]. The concentrations of Cd and As on the surfaces of rice roots in the exogenous Fe treatments were higher (*p* < 0.05) than those in the control, indicating that Cd and As were enriched in the Fe plaque. However, the Fe plaque that formed on the roots surfaces differed owing to the differences in Fe materials, thus, affecting the absorption of Cd and As by rice. This was also the primary reason why the amount of Cd and As adsorbed on the Fe plaque formed by Nano-Fe was higher than that of FeSO_4_. Furthermore, Fe oxide can bind heavy metals in the soil [49], and the plants barely absorb the combined portion of Cd or As and Nano-Fe. The concentrations of Cd and As and uptake in rice roots, shoots, and grains of exogenous Fe treatments were reduced (*p* < 0.05), which indicated that the Fe plaque absorbed Cd and As and prevented these compounds from entering the rice roots.

TF represents the plant’s ability to translocate the pollutant from the roots to the aerial parts of the plant. The TFs _root/soil_ of Cd and As were lower (*p* < 0.05) than those of the control, which indicated that the application of exogenous Fe reduced the ability of Cd and As to enter into the roots from the soil. There are two factors that could lead to this phenomenon. One could be that the addition of exogenous Fe led to a decrease in the amount of available Cd and As in the soil, whereas the other was that Fe plaque prevented the rice from absorbing Cd and As. The TF _shoot/root_ and TF _grain/shoot_ indicated the translocation, which was affected by xylem loading, intravascular transfer, and the transport of heavy metal through the phloem [50,51]. The Cd TFs _shoot/root_ of the Nano-Fe was lower than that of the control and FeSO_4_ (*p* < 0.05) (Figure 4b,c). The Cd TFs _grain/shoot_ of the Nano-Fe compared with the FeSO_4_ treatment, and the Nano-Fe treatment could reduce the ability of Cd to be transferred from the rice roots to shoots, and thus, reduce the accumulation of Cd in rice grains. Compared with the control, exogenous Fe treatments could decrease the TFs _shoot/root_ and TFs _grain/shoot_ of As (Figure 4e,f). The difference in TFs between different treatments could be related to rice genomics or other factors that affect the accumulation of Cd and As in rice, such as translocation in the plant and interactions between heavy metals and other mineral nutrients. Cd was usually absorbed and transported in rice through the transport system of essential cations, such as Ca, Fe, and Mn [52]. These transport systems were regulated by P1B ATPase (heavy metal ATPase), and many genes that encode these transporters are members of the P1B ATPase family [53,54,55]. The genes that regulate the expression of these proteins could be affected by changing environmental factors, such as exogenous Fe and Fe dosage. The Nano-Fe alleviates As phytotoxicity in rice by improving the uptake of Fe, increasing oxidative stress tolerance and diminishing the accumulation of As [43].

### 4.3. Health Risk Index

Rice is the key manner of human exposure to heavy metals [56]. It is very important to quantify the health risk assessment of Cd and As in rice. In this study, although the Cd *HRI* value in the control was <1, the risk of long-term consumption of rice produced in this region cannot be ignored [26]. The addition of exogenous Fe reduced the Cd, As, and Cd-As *HRIs*, which indicated that exogenous Fe could effectively reduce their risk. In particular, the reduction of Nano-Fe was the greatest. A health risk index >1 is considered to be a threat to human health. The *HRIs* for Cd-As of the treatments (FeSO_4_ and Nano-Fe) were <1 and thus, met the safety limits.

## 5. Conclusions

In this study, FeSO_4_ and Nano-Fe that were applied at 0.5 g/kg, and were used to treat paddy soil contaminated with Cd-As to examine their influences on the forms of Cd and As in soil and soil microorganisms, the transport and accumulation of Cd and As in rice, and the risk assessment of human intake. The results demonstrated that the application of exogenous Fe as both forms of FeSO_4_ and Nano-Fe significantly improved the diversity and structure of microbial community, particularly increasing the relative abundances of Cd and As-resistant bacteria, and reducing the contents of available Cd and As in the soil. Moreover, exogeFnous Fe could promote the formation of Fe plaque, reduce the transport and accumulation of Cd and As in rice, and reduce the risk of human intake. The results of our study confirm that supplementation of environmental-friendly Nano-Fe 0.5 g·kg^−1^ to paddy soil is an effective approach to simultaneously reduce Cd and As accumulation in the rice grains grown in co-contaminated soil.

## Figures and Tables

**Table 1:** Effect of exogenous Fe on the yield of various parts of rice.

Treatments	Root Yield (g)	Shoot Yield (g)	Grain Yield (g)
Control	25.66 ± 0.14c	156.07 ± 8.61b	76.33 ± 0.93b
FeSO_4_	26.18 ± 0.03bc	178.20 ± 0.88a	83.33 ± 0.79a
Nano-Fe	28.40 ± 0.45a	191.00 ± 0.43a	83.46 ± 0.22a

Different lowercase letters in the columns indicate a significant difference among the different treatments at *p* < 0.05.

**Table 2:** Effect of exogenous Fe applied as FeSO_4_ and Nano-Fe on the Cd contents in soil.

Treatments	Exchangeable Cd (μg·kg^−1^)	Organic Bound Cd (μg·kg^−1^)	Inorganic Bound Cd (μg·kg^−1^)	Residual Cd (μg·kg^−1^)
Control	6.75 ± 0.06a	19.27 ± 0.06a	1119.67 ± 0.98a	583.33 ± 5.44c
FeSO_4_	6.13 ± 0.01b	13.36 ± 0.18b	1076.67 ± 10.89b	633.33 ± 7.20b
Nano-Fe	3.31 ± 0.09c	6.79 ± 0.26c	965.67 ± 14.03c	753.33 ± 7.20a

Different lowercase letters in the columns indicate a significant difference among the different treatments at *p* < 0.05.

**Table 3:** Effect of exogenous Fe applied as FeSO_4_ and Nano-Fe on As contents in soil.

Treatments	Non-Specifically Sorbed As (mg·kg^−1^)	Specifically Sorbed As (mg·kg^−1^)	Amorphous Iron Oxide As (mg·kg^−1^)	Fe/Al Hydrated Oxide As (mg·kg^−1^)	Residual As (mg·kg^−1^)
Control	0.25 ± 0.00a	12.09 ± 0.05a	49.61 ± 0.24a	28.38 ± 0.14c	16.61 ± 0.14b
FeSO_4_	0.26 ± 0.01a	9.49 ± 0.14b	48.83 ± 0.57ab	30.96 ± 0.25b	17.40 ± 0.62ab
Nano-Fe	0.25 ± 0.01a	8.2 ± 0.13c	48.18 ± 0.31b	32.09 ± 0.36a	18.21 ± 0.12a

Different lowercase letters in the columns indicate a significant difference among the different treatments at *p* < 0.05.

**Table 4:** Effect of exogenous Fe applications on Shannon’s and Simpson’s indices of the bacterial communities.

Treatments	Shannon’s Index	Simpson’s Index
Control	6.4452 ± 0.0058c	0.00536 ± 0.00004a
FeSO_4_	6.4920 ± 0.0078b	0.00518 ± 0.00002b
Nano-Fe	6.6208 ± 0.0124a	0.00416 ± 0.00013c

Different lowercase letters in the columns indicate a significant difference among the different treatments at *p* < 0.05.

**Table 5:** Risk assessment of Cd and As intake via rice grain treated with exogenous Fe.

Treatments	Cd		As		HRI Cd-As
	DIM	HRI	DIM	HRI	
Control	0.00019	0.19	0.00040	1.34	1.53
FeSO_4_	0.00012	0.12	0.00022	0.73	0.85
Nano-Fe	0.00006	0.06	0.00010	0.34	0.40

## Data Availability

The data sets supporting the results of this article are included within the article.

## Supplementary Materials

The following supporting information can be downloaded at: https://www.mdpi.com/article/10.3390/nano12081311/s1, Figure S1: Scanning electron microscopy (SEM) of Nano-Fe; Figure S2: X-ray energy spectrum analysis (EDS) of Nano-Fe; Figure S3: Fourier transform infrared spectroscopy (FTIR) of Nano-Fe.
