# Supplementary material for: Application of Exogenous Iron Alters the Microbial Community Structure and Reduces the Accumulation of Cadmium and Arsenic in Rice (Oryza sativa L.)
Source: Nanomaterials (Basel). 2022 Apr 11;12(8):1311. doi: 10.3390/nano12081311 (PMC9028164; doi:10.3390/nano12081311)
Supplement: Supplementary file 1 [file nanomaterials-12-01311-s001.zip › nanomaterials-1653188-supplementary.pdf]

nanomaterials

Supplementary materials

Application of Exogenous Iron Alters the Microbial Community Structure and Reduces the Accumulation of Cadmium and Arsenic in Rice (*Oryza sativa* L.)

Tingting Li [1,2], Jiayuan Li [1], Xin Zhan [1], Xueli Wang [1] ,Bing He [1], Feishu Cao [3], Changjun Liao [3], Yuefeng Yu [2], Zengyu Zhang [1], Junhui Zhang [2], Bei Li [1], Jiancheng Chen [1], Hong Li [4], Zhiqiang Zhu [5], Yanyan Wei [1,*] and Junming Hu [2,*]

[1] State Key Laboratory for Conservation and Utilization of Subtropical Agro-Bioresources，Cultivation Base of Guangxi Key Laboratory for Agro-Environment and Agro-Products Safety, College of Agriculture, Guangxi University, Nanning 530004, China; ltt1210@126.com (T.L.); 1917303005@st.gxu.edu.cn (J.L.); njueczx@163.com (X.Z.); wxl0524@126.com (X.W.); bingh2000@126.com (B.H.); 1817303017@st.gxu.edu.cn (Z.Z.); le_bei@st.gxu.edu.cn (B.L.); 2017392001@st.gxu.edu.cn (J.C.);
[2] Agricultural Resources and Environment Research Insititute, Guangxi Academy of Agricultural Sciences, Nanning 530007, China; yuyue202204@163.com (Y.Y.); zjh914zjh914@163.com (J.Z.);
[3] Guangxi Bocco Enviromental Protection Technology Co., Ltd., Nanning 530007, China; feishu.cao@hotmail.com (F.C.); lcj19176042408@163.com (C.L.);
[4] Key Laboratory of Eco-Environment of Three Gorges Region, Ministry of Education, Chongqing University, Chongqing 400044, China; hongli@cqu.edu.cn
[5] College of Tropical Crops, Hainan University, Haikou 570228, China; zqzhu@hainanu.edu.cn
* Correspondence: yanyanwei@gxu.edu.cn and yanyanwei2008@163.com (Y.W.); jmhu06@126.com (J.H.); Tel.: +86-1860-7718-450 (Y.W.)

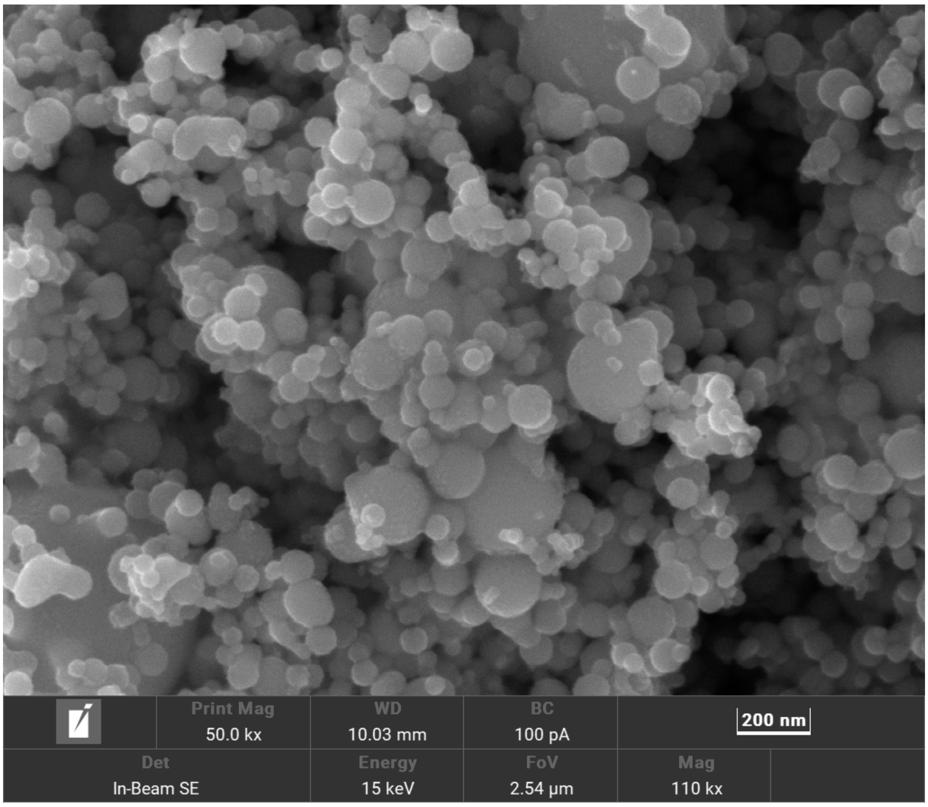

Figure S1. Scanning electron microscopy (SEM) of Nano-Fe.

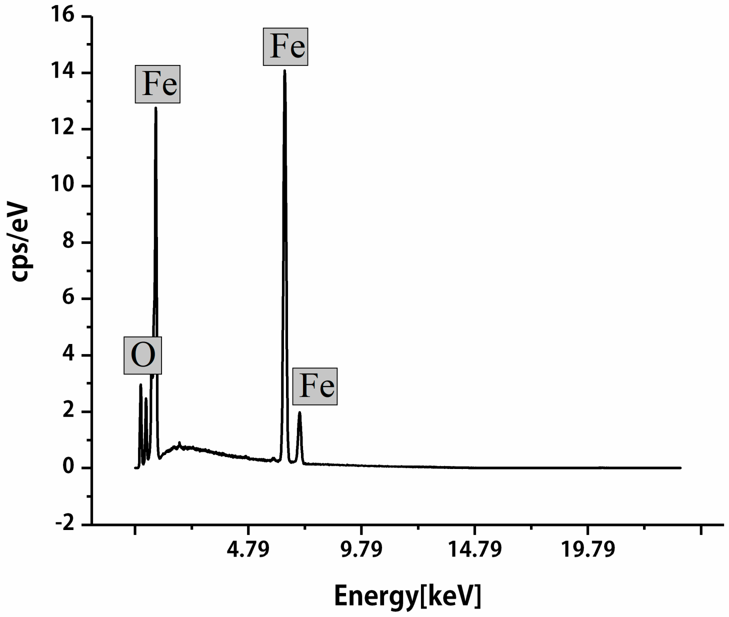

Figure S2. X-ray energy spectrum analysis (EDS) of Nano-Fe.

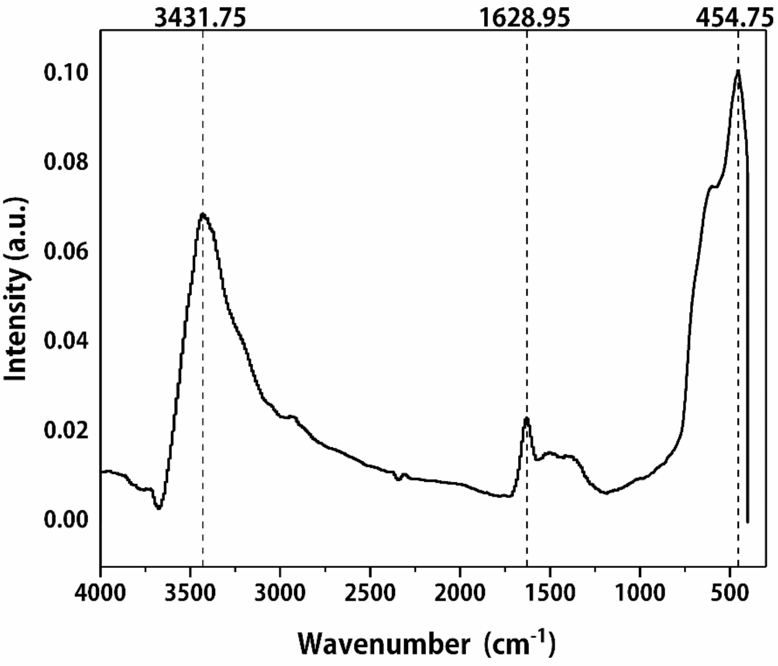

Figure S3. Fourier transform infrared spectroscopy (FTIR) of Nano-Fe.
